# Stroke and Recent Myocardial Infarction, Reduced Left Ventricular Ejection Fraction, Left Ventricular Thrombus, and Wall Motion Abnormalities

**DOI:** 10.1007/s11886-023-02009-y

**Published:** 2023-12-11

**Authors:** Ana Catarina Fonseca

**Affiliations:** 1 Stroke Unit, Department of Neurology, Hospital de Santa Maria, Centro Hospitalar Universitário Lisboa Norte, Avenida Professor Egas Moniz, 1640-035 Lisboa, Portugal; 2 Instituto de Medicina Molecular, Faculdade de Medicina, Universidade de Lisboa, Lisboa, Portugal; 3 Centro de Estudos Egas Moniz, Faculdade de Medicina, Universidade de Lisboa, Lisboa, Portugal

**Keywords:** Stroke, Myocardial infarction, Thrombus, Ejection fraction, Wall motion abnormalities, Heart failure, Thrombolysis, Thrombectomy, Treatment

## Abstract

**Purpose of Review:**

To review the evidence regarding stroke and recent myocardial infarction (MI), reduced left ventricular ejection fraction, left ventricular thrombus (LVT), and wall motion abnormalities (WMA).

**Recent Findings:**

The risk of ischemic stroke associated with acute MI has been greatly reduced with reperfusion treatments that improved myocardium salvage. Acute ischemic stroke is an uncommon complication of diagnostic coronary angiography and percutaneous coronary intervention. For established LVT, anticoagulation is superior to antiplatelet medications to reduce the risk of ischemic stroke. The duration of anticoagulation should be at least 3 to 6 months. Direct oral anticoagulants have been used off-label in this context. In patients with low ejection fraction or WMA, there is no evidence that anticoagulation is superior to antiplatelet treatment in preventing ischemic stroke. In patients with ischemic stroke and recent MI (< 3 months), type of MI (STEMI or NSTEMI), timing, and location should be considered when deciding whether intravenous thrombolysis should be used for stroke treatment. Mechanical thrombectomy should be considered as a therapeutic alternative to intravenous thrombolysis in patients with acute ischemic stroke due to large-vessel occlusion and recent MI.

**Summary:**

Most guidelines regarding prevention of ischemic stroke in patients with these cardiac causes of stroke are derived from expert opinion. There is a need for high quality evidence to support stroke prevention treatments in these patients.

## Introduction

Cardioembolism is a major cause of ischemic stroke and encompasses many other possible mechanisms of stroke in addition to atrial fibrillation (AF) [1]. Cardiac causes of ischemic stroke can be divided into three main groups according to the underlying mechanism: (1) cardiac wall and chamber abnormalities, (2) valve disorders, and (3) arrhythmias [2]. Acute myocardial infarction (MI), reduced left ventricular ejection fraction (LVEF), left ventricular thrombus (LVT), and wall motion abnormalities (WMA) are part of the group of cardioembolic causes of stroke related to cardiac wall and chamber abnormalities.

The embolic risk of these cardiac causes of ischemic stroke differs and is divided into high and medium embolic risk [3]. According to the Trial of Org 10,172 in Acute Stroke Treatment (TOAST) classification which is the most widely used stroke etiological classification system, acute MI (< 4 weeks), LVT and akinetic ventricular segment disease are considered as high-risk sources of cardioembolism, whereas hypokinetic left ventricular segment disease and myocardial infarction (> 4 weeks, < 6 months) are considered medium risk sources of cardioembolism [3].

Knowledge about these cardiac sources of embolism has evolved in recent years. We aim to review key recent findings.

## Epidemiology

### Myocardial Infarction

The risk of ischemic stroke is highest in the first 5 days after acute MI and decreases greatly after the first 4 weeks [4]. Nevertheless, MI can be a cause of acute ischemic stroke, not only in the acute phase, but also in the long term. Aggressive treatment of acute MI, namely by using reperfusion therapies, reduced the risk of ischemic stroke by improving salvage of myocardium at risk and diminishing WMA [5, 6]. An analysis of acute MI admissions in the USA using the National Inpatient Sample database from 2000 to 2017 showed that of a total of 11,622,528 acute MI admissions, 1.6% had concomitant acute ischemic stroke [7]. Compared with 2000, in 2017, rates of acute ischemic stroke increased slightly among ST-elevation MI (STEMI) (adjusted odds ratio, 1.10 [95% confidence interval (CI), 1.04–1.15]) and decreased in Non-STEMI (NSTEMI) (adjusted odds ratio, 0.47 [95% CI, 0.46–0.49]) admissions (*P* < 0.001). Compared to those without stroke, the acute ischemic stroke cohort was on average older, female, of non-white race, with greater comorbidities, and higher rates of arrhythmias [7]. The acute MI-acute ischemic stroke admissions group had received less frequently coronary angiography (46.9% versus 63.8%) and percutaneous coronary intervention (PCI) (22.7% versus 41.8%) (*P* < 0.001) [7].

### Left Ventricular Thrombus

The risk of LVT formation after MI is high in the first 3 months after acute MI but especially in the first 2 weeks [6]. STEMI patients are more likely to have LVT compared to NSTEMI. Recent anterior transmural myocardial infarctions are often associated with the finding of LVT. The incidence of LVT after anterior STEMI varies in different studies, from 4 to 39% which reflects different cohorts, diagnostic methods, timing, and frequency of screening [8••]. There is some evidence that the incidence of LVT has decreased over time due to improved reperfusion interventions [5, 6]. Predictors of LVT include anterior MI, involvement of left ventricular apex, WMA, reduced LVEF, severe diastolic dysfunction, large myocardial infarct size, and spontaneous echo-contrast [5, 9]. Delaying imaging for ≥ 5 days after STEMI is associated with a substantial increase in LVT detection rates, reaching a peak at 9–12 days [10].

LVT can occur not only after an acute MI but also in ischemic and non-ischemic cardiomyopathies. LVT incidence in non-ischemic cardiomyopathies varies between 2 and 36% [11]. In the Warfarin and Antiplatelet Therapy in Chronic Heart Failure (WATCH) trial that evaluated patients with chronic dilated cardiomyopathy in sinus rhythm, predictors of LVT were younger age, lower ejection fraction, higher regional wall motion score, higher early diastolic filling velocity, shorter deceleration time, greater left ventricular diastolic dimension, and left atrium area [11].

### Wall Motion Abnormalities and Reduced Left Ventricular Ejection Fraction

The main cause of cardiac WMA is MI. In a 1995 Icelandic population-based prospective cohort study, at least one-third of all MI was unrecognized [12]. This number may currently be lower due to increased public awareness of the symptoms and signs of acute MI and improved diagnosis. However, it is important to consider that an history of MI is not always present in patients with WMA and that undetected MI can also lead to left ventricular scarring and/or WMAs [13]. LVT can develop months after MI and overlie ventricle wall segments with abnormal motion that can embolize [13].

Reduced LVEF is a risk factor for ischemic stroke. The risk of stroke is inversely correlated with LVEF in patients with sinus rhythm [14].

Reduced LVEF, WMA, and LVT can be screened by transthoracic echocardiogram (TTE). Canadian Best Practice Guidelines recommend the routine use of TTE in the initial workup of ischemic stroke patients when an embolic source is suspected [15]. The 2019 American Heart Association/American Stroke association (AHA/ASA) guidelines update states that for prevention of recurrent stroke, the use of echocardiography is reasonable in some patients with acute ischemic stroke to provide additional information to guide selection of appropriate secondary stroke prevention (IIa, C-EO) [16]. Contrast echocardiography with the use of a microbubble contrast agent and cardiac magnetic resonance imaging (CMR) are superior imaging modalities for detecting LVT compared to standard TTE [17, 18]. Adapting the imaging strategy based on patient risk can optimize diagnostic yield.

Currently, there is no recommendation to perform transesophageal echocardiogram (TEE) to study ischemic stroke etiology in patients with acute MI [16]. In patients with AF complicating acute MI, in whom AF/left atrial appendage thromboembolism can cause ischemic stroke, it is considered that these patients will benefit from oral anticoagulation regardless of echocardiographic findings [16].

## Associated Mechanisms and Risk of Stroke

Patients with acute MI are at increased risk of ischemic stroke due to the presence of factors that compose the “Virchow triad” (tissue injury, blood stasis, and hypercoagulability). In these patients, acute ischemia can lead to endomyocardial injury, blood stasis (due to reduced ventricular contractility, wall motion abnormalities particularly apical, and/or severe diastolic dysfunction) and hypercoagulability (proinflammatory state post-MI) that increase the likelihood of left ventricular thrombosis. Patients with STEMI infarcts with anterior wall involvement are at particular high risk [19].

More rarely, ischemic and hemorrhagic strokes can also result from a complication of treatment for acute MI. Fibrinolytic therapy for MI treatment is associated with a small but significant increase in strokes, essentially due to an increase in cerebral hemorrhage on the first day after treatment [20]. Acute ischemic stroke is an uncommon complication of diagnostic coronary angiography (DCA) and PCI [21]. Periprocedural stroke occurs in 0.05–0.1% of DCA cases [22]. The incidence of post-PCI ischemic stroke varies between different studies. In single-center registries, the incidence of post-PCI ischemic stroke ranged from 0.37 to1.3% [21]. In randomized controlled trials (RCT), the incidence of post-PCI stroke was 0.8 to 1.4% with PCI for acute MI and 0.4 to 0.8% with PCI for unstable angina or stable ischemic heart disease [21]. Most strokes related to DCA and PCI are embolic, due to dislodgement of a clot or atheromatous debris from the aortic arch or thrombus formation in the guide catheter [23, 24]. Carotid artery disease, cardiogenic shock, AF, renal failure, femoral access, female gender, and older age are the strongest predictors of periprocedural ischemic stroke [21, 24]. Khatibzadeh et al. showed that atherosclerotic plaques prone to dislodgement in the thoracic aorta (descending and arch) predispose to ischemic stroke in patients treated from a femoral access [25]. TEE is the modality of choice to evaluate aortic plaque, complex atheroma (plaques with > 4-mm thickness or with plaque rupture and mobile fragments, and ulcerated plaques) which are more likely to be associated with embolic events in the context of periprocedural stroke [26]. However, there is currently no formal recommendation to perform TEE in the setting of periprocedural stroke [21]. Low institutional annual PCI volume is also independently associated with increased risk of post-PCI stroke [21].

A safety concern emerged in the Trial of Routine Aspiration Thrombectomy with PCI versus PCI Alone in Patients with STEMI (TOTAL) trial, with an increase in the risk of ischemic stroke [27]. In the subgroup with high thrombus burden [TIMI (thrombolysis in myocardial infarction) thrombus grade ≥ 3], thrombus aspiration was associated with more ischemic strokes or transient ischemic attacks (TIAs) [55 (0.9%) vs 34 (0.5%); odds ratio 1.56, 95% CI 1.02–2.42, *P* = 0.04) [27, 28, 29••]. In these cases, an incompletely aspirated thrombus may have dislodged inside the guide catheter to be subsequently injected into the systemic circulation where it embolized [30]. Furthermore, in STEMI patients treated with PCI, new-onset acute ischemic stroke or TIA was significantly associated with cardiogenic shock, new-onset AF, transfemoral approach, use of ≥ 4 catheters, and Bleeding Academic Research Consortium (BARC) type 3 or 5 bleeding [19]. These risk factors for acute ischemic stroke/TIA should be identified in patients treated for STEMI [19]. Manipulation of the ascending aorta during on-pump coronary artery bypass grafting (CABG) surgery can release embolic matter and cause ischemic stroke [31].

The presence of LVT after acute MI is associated with a 5.5-fold increased risk of embolic events compared to the absence of thrombus [32]. In the past, LVT has been associated with up to a 22% risk of embolization and a 37% risk of major adverse cardiovascular events [8••]. Patients with LVEF < 50% in the context of acute anterior MI are at increased risk of developing LVT [33]. Large areas of infarct increase the risk of thrombus formation. Protruding, pedunculated, and mobile thrombi are more likely to embolize than immobile, calcified, and laminated thrombi [34]. LVT size rarely appears as a risk factor for embolization, and when it does appear, it is a lesser risk factor [8••].

The risk of ischemic stroke is greatest in the acute phase after MI but persists thereafter.

A history of coronary artery disease may be a clue for the presence of WMA disease such as segmental akinesia or hypokinesia. In a retrospective analysis of prospectively collected acute stroke data from a total of 2653 patients with acute ischemic stroke/TIA, WMA was observed in 355 patients (13.4%) [13]. In this cohort of ischemic stroke patients with WMA, WMA independently conferred an increased risk of ischemic stroke recurrence (adjusted HR, 1.50; 95% CI, 1.01–2.17 [*P* = 0.04]), risk of acute coronary events (aHR, 2.50; 95% CI, 1.83–3.40 [*P* = 0.001]) and vascular death (adjusted HR, 1.57; 95% CI, 1.04–2.40 [*P* = 0.03 [13]. WMAs were found to an independent predictor when controlling for ejection fraction [13].

Ejection fraction can be preserved or recover post-MI, particularly in small MIs, this may happen because the hypercontractile left ventricular segments compensate for the dysfunctional segment. Heart failure with reduced ejection fraction with left ventricular ejection fraction < 40% is increasingly associated with an increased risk of ischemic stroke, even in the absence of AF [13]. This can be due to endothelial dysfunction and intra-ventricular stasis leading to microthrombi formation [13].

## Ischemic Stroke Prevention

### Prevention of Ischemic Stroke in Patients with Acute MI

Aggressive treatment of acute MI, namely using reperfusion therapies and dual antiplatelet therapy (DAPT), reduced the risk of ischemic stroke by improving salvage of myocardium at risk [5, 6]. Evidence from clinical trials showed that if treatment delay is similar, primary PCI is superior to fibrinolysis in reducing stroke [29••]. Due to the data from the TOTAL trial and the results of a meta-analysis [27, 28], routine thrombus aspiration is not recommended [29••, 35]. Nevertheless, the European Society of Cardiology (ESC) guidelines state that in cases of large residual thrombus burden after opening the vessel with a guide wire or a balloon, thrombus aspiration may be considered [29••].

Because CABG has been associated with an increased risk of ischemic stroke, some authors suggest that avoiding aortic clamping may be important in decreasing CABG-related stroke rates [31]. In a recent review of patients treated with CABG, a hybrid strategy incorporating off-pump, pump-assisted, and combined off-pump/pump-assisted techniques achieved very low ischemic stroke rates in patients undergoing coronary revascularization [31].

The 2021 AHA/ASA guidelines for the Prevention of Stroke in Patients with Stroke and TIA state that in patients with ischemic stroke or TIA in the setting of acute MI, it is reasonable to perform advanced cardiac imaging (e.g., contrasted echocardiogram or CMR) to assess the presence of LVT (2a, C-EO) [16].

The European Society of Cardiology STEMI treatment guidelines recommend the use of routine echocardiography to assess resting left ventricular and right ventricular function, detect early post-MI mechanical complications, and exclude LVT in all STEMI patients (I,B) [29••].

ESC guidelines also state that routine post-procedural anticoagulant therapy is not indicated after primary PCI, except when there is a separate indication for full-dose anticoagulation (due, for example, to AF, mechanical valves, or LVT) [29••].

Recently, a multicenter, retrospective cohort study was performed to evaluate the comparative effectiveness and safety of DAPT vs triple therapy (TT) with DAPT + oral anticoagulant (OAC) in patients with anterior STEMI and with new-onset anterior/apical WMAs treated with PCI [36]. A total of 1666 patients were included. The addition of OAC to DAPT in anterior STEMI patients with new-onset WMA treated with PCI was not associated with a significant reduction in a composite endpoint at 6 months of all-cause mortality, nonfatal MI, stroke, or TIA, systemic thromboembolism or type 3 or 5 BARC bleeding [36].

Prasugrel and ticagrelor, which are P2Y_12_ inhibitors and can be used after acute MI, are contraindicated in patients with previous stroke [29••]. Prasugrel is contraindicated in patients with previous stroke/TIA [37, 38], while ticagrelor is contraindicated in patients with a previous intracranial hemorrhage (ICH) because of a high risk of recurrent ICH in this population [39, 40]

Prasugrel is contraindicated in patients with previous stroke/TIA due to the results of the TRITON-TIMI 38 (TRial to Assess Improvement in Therapeutic Outcomes by Optimizing Platelet InhibitioN with Prasugrel) [38, 41]. In this trial, patients with a history of TIA or ischemic stroke (> 3 months prior to enrollment) had a higher rate of stroke on prasugrel (6.5%; of which 4.2% were thrombotic stroke and 2.3% were ICH) than on clopidogrel (1.2%; all thrombotic) [41]. In patients without such a history, the incidence of stroke was 0.9% (0.2% ICH) and 1.0% (0.3% ICH) with prasugrel and clopidogrel, respectively [41].

### Stroke Prevention in Patients with LVT

Scarce evidence exists regarding optimal antithrombotic therapy and duration in patients with LVT. Recommendations from guidelines and scientific societies are often based on consensus or expert opinions.

A small RCT suggested that anticoagulant therapy is more likely to resolve LVT and to lower embolic risk compared with no warfarin or antiplatelet therapy (60% versus 10%; *P* < 0.01) [42]. These results were reproduced in a meta-analysis of 7 observational studies [32].

In the presence of a LVT, there is no randomized evidence available to compare different antithrombotic regimens after an acute MI, such as TT consisting of an OAC plus DAPT versus double (dual) therapy of a single antiplatelet with OAC [10, 43••, 44]. The AHA scientific statement for the management of LVT states then when OAC is added to antiplatelet therapy in patients who have undergone PCI, a general strategy of double therapy, with OAC (preferably a direct oral anticoagulant) and a P2Y_12_ inhibitor (preferably clopidogrel), after 1 to 4 weeks of triple therapy‚ is preferred over longer-term triple therapy consisting of an OAC plus DAPT [8••].

There is also little evidence available regarding the optimal duration of anticoagulation in patients with LVT after MI. There are data suggesting that LVT should be considered a marker of increased long-term thrombotic risk that persists even after treatment and documented resolution of LVT on imaging [34, 35]. Currently, AHA/ASA guidelines recommend that in patients with ischemic stroke or TIA and LVT, anticoagulation with therapeutic warfarin with an INR goal of 2.0 to 3.0 for at least 3 months is recommended to reduce the risk of recurrent stroke [8••, 17]. If there is thrombus resolution it is considered reasonable to discontinue OAC after 3 months. Earlier stopping of anticoagulation even with resolution of the thrombus is not advised if the left ventricular function impairment or WMA remains (akinesia or dyskinesia) [8••]. ESC guidelines indicate that in patients with LVT, anticoagulation should be administered for up to 6 months guided by repeated imaging (Class: IIa, L:C) [29••]. The LEVITATION survey showed that some centers use direct oral anticoagulants (DOAC) as the anticoagulant therapy of choice despite it being an off-label indication [10]. A meta-analysis that included 39 studies (29 retrospective cohort studies, 4 prospective cohort studies, 3 post hoc analyses or subgroup analyses of a RCT, 1 RCT, 1 cross-sectional study and 1 cohort of unclear design) with 5475 patients with LVT (1953 with known prior MI) showed that in patients with LVT, DOACs were associated with reduced mortality [RR, 0.66; 95% CI, 0.45–0.97; *I*^2^ = 9%] and bleeding (RR, 0.64; 95% CI, 0.48–0.85; *I*^2^ = 0%) compared with warfarin but there was a nonsignificant reduction in strokes/embolic events (RR, 0.95; 95% CI, 0.76–1.19; *I*^2^ = 3%) [45]. The average mean age of patients included in the meta-analysis was 59 years, and the average proportion of male patients was 75%.

The choice of a specific DOACs is also debatable. In this meta-analysis, apixaban was the OAC associated with the highest rate of thrombus resolution (93.3%) while warfarin exhibited the lowest rate (73.1%) [45].

There also is little evidence on the duration of anticoagulation in patients with LVT and nonrecent MI. The AHA scientific statement recommends that anticoagulation with a vitamin K antagonist (VKA) or DOAC should be done in these patients for at least 3 to 6 months [8••]. A shared decision-making approach is also recommended to decide whether anticoagulation should be continued indefinitely considering factors such as lack of improvement in left ventricular systolic function, bleeding risk, tolerability of OAC, and the patient’s risk of possible stroke or bleeding complications [8••].

In case of a LVT in the context of a dilated cardiomyopathy, the AHA based on retrospective registry data and small, prospective observational studies, suggests anticoagulation (VKA or DOAC) in patients with LVT for at least 3 to 6 months, with discontinuation if LVEF improves to > 35% (assuming resolution of the LVT) or if major bleeding occurs [8••]. There are no data on indefinite anticoagulation, for patients in whom left ventricular systolic function does not improve with guideline-directed therapies, in those who have persistent apical akinesia or dyskinesis, and in patients with patients with proinflammatory or hypercoagulable states such as malignancy or renal failure [8••].

There are only a few anecdotal reports and retrospective small case series of surgical excision of LVT. Surgical removal of LVT may be considered in the presence of a very high risk of embolism, nevertheless there is very little data, particularly on the outcomes [8••, 46].

### Prevention of Ischemic Stroke in Patients with Reduced LVEF or WMA

In patients with ischemic stroke or TIA in sinus rhythm with ischemic or non-ischemic cardiomyopathy and reduced LVEF without evidence of left atrial thrombus or LVT, the effectiveness of anticoagulation compared with antiplatelet therapy is uncertain.

Several RCTs evaluated the use of anticoagulation versus antiplatelet treatment in patients with reduced LVEF in sinus rhythm [4, 47–50]. In these trials, stroke was either a primary outcome or part of a composite outcome. Overall, there was no significant difference in treatment between anticoagulation and aspirin. In some trials, anticoagulation was found to be associated with an increase in major bleedings that surpassed a potential benefit in stroke reduction [4]. Table 1 summarizes the main characteristics and results of these trials. Therefore, available evidence does not support the routine use of anticoagulation in patients with heart failure and reduced LVEF who remain in sinus rhythm.

**Table 1:** Summary of randomized clinical trials that evaluated the use of anticoagulation versus antiplatelet treatment in patients with reduced left ventricular ejection fraction/heart failure in sinus rhythm

Name of clinical trial	Inclusion criteria	Treatment	Primary outcome	Number of patients	Mean follow-up	Result primary outcome	Stroke	Major hemorraghe
WARCEF	LVEF < 35%	Warfarin (INR 2.0–3.5) versus aspirin	Time to the first event in a composite end point of ischemic stroke, intracerebral hemorrhage, or death from any cause	2860	6 years	7.47 events per 100 patient-years in the warfarin group and 7.93 in the aspirin group (hazard ratio with warfarin, 0.93; 95% CI 0.79 to 1.10; *P* = 0.40)	Warfarin, as compared with aspirin, was associated with a significant reduction in the rate of ischemic stroke throughout the follow-up period (0.72 events per 100 patient-years vs. 1.36 per 100 patient-years; hazard ratio, 0.52; 95% CI, 0.33 to 0.82; *P* = 0.005)	The rate of major hemorrhage was 1.78 events per 100 patient-years in the warfarin group as compared with 0.87 in the aspirin group (*P* < 0.001).
COMMANDER HF	LVEF ≤ 40% and coronary heart disease, NT-proBNP ≥ 800 pp per ml or BNP ≥ 200 pp per ml	Rivaroxaban at a dose of 2.5 mg twice daily versus placebo	Composite of death from any cause, myocardial infarction, or stroke	5022	21.1 months	Primary end point occurred in 626 (25.0%) of 2507 patients assigned to rivaroxaban and in 658 (26.2%) of 2515 patients assigned to placebo (hazard ratio, 0.94; 95% confidence interval [CI], 0.84 to 1.05; *P* = 0.27)	The rates of stroke were 2.0% and 3.0%, in the rivaroxaban and warfarin groups respectively (hazard ratio, 0.66; 95% CI, 0.47 to 0.95)	18 patients (0.7%) assigned to rivaroxaban and 23 (0.9%) assigned to placebo (hazard ratio, 0.80; 95% CI, 0.43 to 1.49; *P* = 0.48).
HELAS	Symptomatic HF, in NYHA class II-IV and an ejection fraction < 35%	Warfarin versus placebo and aspirin versus warfarin	Composite endpoint of nonfatal stroke, peripheral or pulmonary embolism, myocardial (re)infarction, re-hospitalization, exacerbation of heart failure, or death from any cause	197	312 patient years of follow-up	The study results showed no significant difference in the incidence of thromboembolism between groups	Only five strokes were recorded. Due to the small number of patients enrolled, it was not possible to evaluate differences in efficacy between the treatment groups	Major hemorrhage only occurred in the warfarin groups, at a rate of 4.6 per 100 patient years.
WASH	Clinical diagnosis of heart failure requiring treatment with diuretics, evidence of left ventricular systolic dysfunction on echocardiography, Echocardiographic criteria of left ventricular dysfunction included an increased left ventricular end-diastolic internal dimension (≥ 56 mm or ≥ 30 mm/m^2^ body surface area) combined with a fractional shortening of ≤ 28% or an echocardiographic ejection fraction ≤ 35%	Warfarin versus aspirin	Composite outcome of death, nonfatal myocardial infarction, and nonfatal stroke	279	27 ± 1 months	No significant difference between the groups in the composite end point of death, stroke, or MI	No significant difference between the groups in stroke	Five major hemorrhagic events occurred. One on aspirin, 2 on warfarin, 1 in a patient who had been withdrawn from warfarin because of a minor bleeding episode 2 years before the event, and 1 who had been withdrawn from warfarin 2 weeks before the event because of the development of a contraindication.
WATCH	Symptomatic heart failure for at least 3 months who were in sinus rhythm and had left ventricular ejection fraction of ≤ = 35%	Warfarin, aspirin, or clopidogrel	Time to first occurrence of death, nonfatal myocardial infarction, or nonfatal stroke	1587	1.9 years (median, 21 months)	Terminated early as a result of failure to reach target enrollment No significant difference among groups in the composite end point of death, nonfatal MI, or nonfatal stroke was observed in those who had been enrolled	Warfarin was associated with fewer nonfatal strokes than aspirin or clopidogrel	Major bleeding episodes were more frequent in warfarin patients compared with clopidogrel (*P* < 0.01) but not aspirin (*P* = 0.22) patients.

*WARCEF *Warfarin versus Aspirin in Reduced Cardiac Ejection Fraction, *HELAS* Heart Failure Long-Term Antithrombotic Study, *WASH* Warfarin/Aspirin Study in Heart Failure, *WATCH* Warfarin and Antiplatelet Therapy in Chronic Heart Failure, *CI* confidence interval, HF heart failure, *INR* International Normalized Ratio, *LVEF* left ventricular ejection fraction, *MI* myocardial infarction, *NYHA* New York Heart Association

Although there are no data to support the routine use of oral anticoagulation for the primary prevention of LVT in patients with reduced LVEF in sinus rhythm, the AHA scientific statement recommends that, on a case-by-case basis, oral anticoagulation maybe considered in patients with specific increased risk for LVT formation such as those with takotsubo syndrome, left ventricular noncompaction, eosinophilic myocarditis, peripartum cardiomyopathy, and cardiac amyloidosis [8••]. In such cases where oral anticoagulation may be implemented, the recommended duration of preventive OAC is not established [8••].

## Treatment of Acute Ischemic Stroke in Patients with Recent Acute Myocardial Infarction

### Intravenous Thrombolysis

Although acute MI in the three months prior to an acute ischemic stroke was not an exclusion criterion in the RCTs evaluating intravenous thrombolysis (IVT) in acute ischemic stroke, there are few data on the safety of IVT in these patients. Some case reports described myocardial rupture, cardiac tamponade, and embolization of ventricular thrombus after IVT for acute ischemic stroke in patients with recent MI [51•]. A systematic review of clinical cases that included 102 patients with recent MI (< 3 months) and acute ischemic stroke reported that four (8.5%) of 47 IVT-treated patients died from confirmed or presumed cardiac rupture/tamponade, all with sub-acute STEMIs in the week preceding stroke [51•]. This complication occurred in 1 (1.8%) patient in the non-treated group. No NSTEMI patients that received IVT experienced cardiac complications [51•]

NSTEMI patients have a lower risk of complications than transmural STEMI patients, and among STEMIs, infarctions in the anterior wall have the highest rates of cardiac complications [52]. Cardiac complications tend to peak 2 to 14 days after a MI. Taking this information into account, the guidelines issued by the ESO and ASA/AHAs divide their recommendations regarding the use of IVT in patients with acute ischemic stroke and recent MI (< 3 months) according to the type (NSTEMI versus STEMI), location, and timing of MI [16, 53].

In case of acute ischemic stroke complicating a concomitant acute MI, guidelines issued by the ESO and ASA/AHA recommend that alteplase may be administered if there are no other contraindications to IVT followed by PCI and stenting if indicated. In this case, ESO guidelines recommend that the dose of alteplase and the time window should conform to the recommendations of IVT for acute ischemic stroke to minimize the risk of symptomatic intracranial hemorrhage [53]. IVT with alteplase with a total dose based on patient weight, not to exceed 100 mg, regardless of the selected administration regimen (accelerated or 3 h) is indicated in patients with acute MI, while in acute ischemic stroke the dose used is 0.9 mg/kg (maximum 90 mg, < 4.5 h) [53].

ESO guidelines suggested not using IVT in patients with acute ischemic stroke of < 4.5-h duration and with history of sub-acute (> 6 h) STEMI during the last seven days (quality of evidence: very low, strength of recommendation: weak). For patients with acute ischemic stroke of < 4.5-h duration and with history of STEMI of more than a week to 3 months, ESO guidelines state that there is insufficient evidence to make a recommendation [53].

The AHA/ASA guidelines are slightly different and recommend that for patients presenting with acute ischemic stroke and a history of recent MI in the past 3 months, treating the ischemic stroke with IV alteplase is reasonable if the recent MI was a STEMI involving the right or inferior myocardium (IIa, C-LD) [16]. In these guidelines, for patients presenting with acute ischemic stroke and a history of recent MI, treating the ischemic stroke with IVT may be reasonable if the recent MI was a STEMI involving the left anterior myocardium (IIb; C-LD) [16].

For patients with acute ischemic stroke and with a history of NSTEMI during the last 3 months, ESO and AHA/ASA suggest intravenous thrombolysis with alteplase (quality of evidence: very low, strength of recommendation: weak; IIa; C-LD respectively) [16, 53].

Intravenous thrombolysis has been suggested as a potential cause of MI after acute ischemic stroke, with some RCTs of IVT for acute ischemic stroke treatment showing a higher number of cardiac events within the thrombolysis group [54, 55]. The proposed mechanism linking IVT to MI was the fragmentation and embolization of an intracardiac thrombus. A case series and systematic review that included patients with acute ischemic stroke who suffered a MI less than 24 h after IVT analyzed this question [56]. This study included fifty-two patients with acute ischemic stroke who suffered a MI less than 24 h after IVT; thirty-two patients (61.5%) were derived from hospital cases. After reviewing 6958 patients treated with IVT [0.5% (95% CI 0.380.54) of total hospital cases], the authors concluded that MI in the first 24 h after IVT for acute ischemic stroke was an infrequent event and more frequently non-embolic [56]. There was an association between the pathophysiology of acute ischemic stroke and MI. However, the prevalence of embolic MI was higher than that found in the general population with MI. The low number of events and publication bias may have limited the study´s conclusions [56].

#### Mechanical Thrombectomy

Patients ineligible for IVT due to medical complications may receive primary mechanical thrombectomy [51•]. Mechanical thrombectomy has been shown to have good results in patients with contraindications for IVT [57, 58]; however, not all patients fulfill the criteria for mechanical thrombectomy (e.g., proximal vessel occlusion), and there is no evidence that primary mechanical thrombectomy has the same results as bridging therapy [51•, 59]. Nevertheless, in patients with acute ischemic stroke after recent STEMI and large-vessel occlusion, primary mechanical thrombectomy may be considered. ESO guidelines state that mechanical thrombectomy should be considered a therapeutic alternative to intravenous thrombolysis in patients with acute ischemic stroke due to large-vessel occlusion and recent MI [53].

## Treatment of Acute Ischemic Stroke in Patients with LVT

There are few cases reported in literature of IVT for acute ischemic stroke in patients with known LVT [9]. The AHA/ASA guidelines state that for patients with major acute ischemic stroke likely to produce severe disability and known LVT, treatment with IVT may be reasonable (IIb; C-LD), but for patients presenting with moderate acute ischemic stroke likely to produce mild disability and known LVT, treatment with IVT is of uncertain net benefit (COR IIb; LOE C-LD) [16]. There is no mention in the ESO guidelines on this topic [16].

## Conclusions

The risk of ischemic stroke after acute MI is well characterized and has decreased over time following the use of reperfusion therapies. Most guidelines from scientific societies that guide primary and secondary prevention of ischemic stroke in these patients derive from expert opinions or consensus. There is a need for close cooperation between cardiologists and stroke physicians to develop high quality evidence namely clinical trials to support ischemic stroke prevention treatments in these patients.
